# The biology of tardigrade disordered proteins in extreme stress tolerance

**DOI:** 10.1186/s12964-020-00670-2

**Published:** 2020-11-04

**Authors:** Cherie Hesgrove, Thomas C. Boothby

**Affiliations:** Department of Molecular Biology, University of Wyoming, Laramie, WY 82071 USA

**Keywords:** Tardigrade, Intrinsically disordered proteins, Tardigrade disordered protein, Cytoplasmic abundant heat soluble protein, Secreted abundant heat soluble protein, Mitochondrial abundant heat soluble protein, Late embryogenesis abundant protein, Anhydrobiosis, Vitrification, Stress tolerance

## Abstract

**Abstract:**

Disordered proteins have long been known to help mediate tolerance to different abiotic stresses including freezing, osmotic stress, high temperatures, and desiccation in a diverse set of organisms. Recently, three novel families of intrinsically disordered proteins were identified in tardigrades, microscopic animals capable of surviving a battery of environmental extremes. These three families include the Cytoplasmic-, Secreted-, and Mitochondrial- Abundant Heat Soluble (CAHS, SAHS, and MAHS) proteins, which are collectively termed Tardigrade Disordered Proteins (TDPs). At the level of sequence conservation TDPs are unique to tardigrades, and beyond their high degree of disorder the CAHS, SAHS, and MAHS families do not resemble one another. All three families are either highly expressed constitutively, or significantly enriched in response to desiccation. In vivo, ex vivo, and in vitro experiments indicate functional roles for members of each TDP family in mitigating cellular perturbations induced by various abiotic stresses. What is currently lacking is a comprehensive and holistic understanding of the fundamental mechanisms by which TDPs function, and the properties of TDPs that allow them to function via those mechanisms. A quantitative and systematic approach is needed to identify precisely what cellular damage TDPs work to prevent, what sequence features are important for these functions, and how those sequence features contribute to the underlying mechanisms of protection. Such an approach will inform us not only about these fascinating proteins, but will also provide insights into how the sequence of a disordered protein can dictate its functional, structural, and dynamic properties.

**Video Abstract**

**Graphical abstract:**

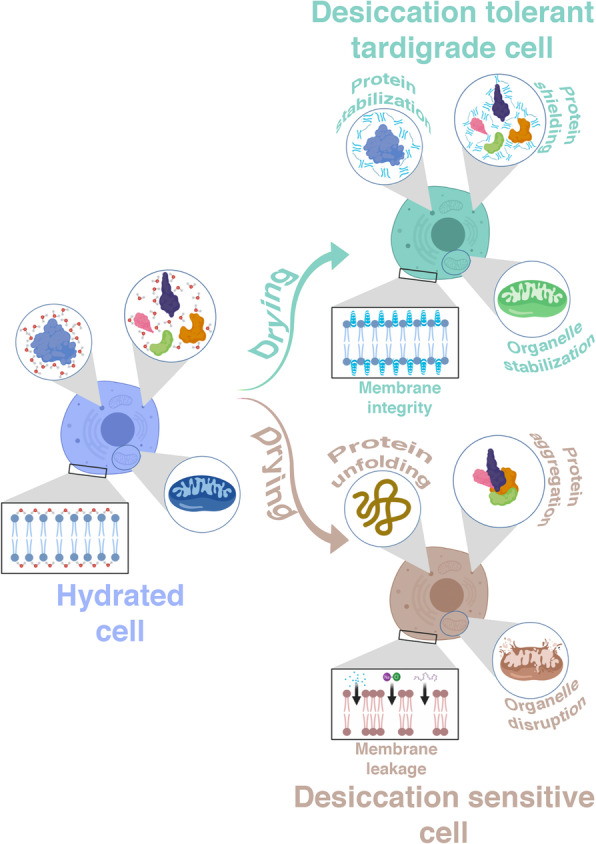

## Plain English summary

Tardigrades (AKA water bears) are a group of microscopic animals capable of surviving a number of environmental extremes such as near complete desiccation, freezing, high temperatures, ionizing radiation, osmotic shock, and extremes in pressure as drastic as the vacuum of outer space. How these diminutive animals are able to survive these stresses, which are typically thought of as being incompatible with life, has remained relatively mysterious since their discovery more than 250 years ago. Recently three novel protein families, which have not been found in other organisms, were discovered in tardigrades. These three protein families are collectively referred to as tardigrade disordered proteins (TDPs). Unlike typical folded proteins, whose function is dictated by their three-dimensional structure, TDPs do not exist in static conformations in solution. Functional experiments have shown that many tardigrade disordered proteins are necessary and sufficient to mediate both natural and induced tolerance to several forms of environmental stress. How the sequence of these unstructured proteins dictates their function(s) is not understood, nor are the mechanism(s) by which these functions are mediated. Experiments addressing these points will not only shed light on how diverse forms of environmental stress are mediated in tardigrades, but also help to understand how the sequences of disordered proteins encode their functional, structural, and dynamic properties under changing environments.

## Background

More than 300 years ago the father of microscopy, Antonie van Leeuwenhoek, added water to a dusty substance he had gathered from a gutter and dried over the course of a summer. In a letter he remarked, “I did not think that any living creature would be present in such a dried-up substance … but I erred in this … after about 1 hour I saw at least a hundred animalcules sitting against the glass as well as running along, and swimming [1].” van Leeuwenhoek had made the first recorded observation of animal anhydrobiosis, a term from Greek meaning ‘life without water.’ Anhydrobiosis, or desiccation tolerance, had been observed even before van Leeuwenhoek peered through his microscope. Nearly every land plant is desiccation tolerant at one or more life stages; seeds, spores, and pollen, to name a few. Today we know of organisms spanning every kingdom of life that tolerate losing most of their intra- and extracellular water [2]. Despite being widespread, in some kingdoms of life anhydrobiosis is a rare trait. The Kingdom Animalia for example, has only four phyla in which anhydrobiosis has been observed: Arthropoda, Nematoda, Rotifera, and Tardigrada.

The phylum Tardigrada consists exclusively of small segmented animals known as tardigrades, or more colloquially as water bears. Despite being some of the smallest animals, ranging in size from 50 to 1000 μm, tardigrades are renowned for their hardiness and resilience to environmental extremes [3]. Among other abiotic stresses, many tardigrade species are capable of surviving: desiccation, high temperature, freezing, osmotic shock, radiation, and even the vacuum of outer space [3]. In some cases, these extreme stress tolerances appear to be inter-dependent. For example, to survive high temperatures or the vacuum of outer space, tardigrades must first be in a desiccated state [4]. The idea of desiccation as a means of preserving or stabilizing biological material, sometimes referred to as xeroprotection, has been pursued in the biomedical field for decades [5–8]. However, the mechanisms underlying anhydrobiosis remain one of the enduring mysteries of physiology. Understanding how anhydrobiosis is naturally mediated promises to accelerate the development of dry storage as a biotechnology. Dry storage as an efficient means of xeroprotection would achieve a long-sought alternative to cryopreservation, breaking humanity’s reliance on refrigeration for medical products ranging from vaccines to insulin and some chemotherapeutics. Thus, understanding how desiccation tolerant organisms naturally survive near complete water-loss is of immense intellectual interest and practical importance.

Works by Jim Clegg, John and Lois Crowe, and more recently Hugo Tapia, Doug Koshland, Takashi Okuda, Chihan Erkut, and Teymuras Kurzchalia as well as myriad others, defined the disaccharide trehalose as a functional mediator of desiccation tolerance [9–13]. Trehalose is both necessary and sufficient to protect a variety of macromolecules, cells, and whole organisms during drying and long-term anhydrobiosis. For a time, it seemed that the mystery of how organisms survive desiccation could be explained primarily through the accumulation of large quantities of trehalose or other disaccharides. However, some very good anhydrobiotes, such as rotifers (small aquatic and highly stress-tolerant animals) and tardigrades, are unlikely to accumulate large quantities of, or even be capable of making, trehalose [14–19]. Tunnacliffe and colleagues presented a series of reports in the early 2000’s demonstrating that Bdelloid rotifers, a group of animals with remarkable stress tolerant abilities including anhydrobiosis, do not accumulate or even possess the genes required to make trehalose [14, 15]. Later, it was shown that some species of tardigrades have low levels of trehalose, while in other species this sugar could not be detected at all [17–19]. At least some tardigrade species appear to lack the canonical genes required to make trehalose, but do possess the genes allowing them to break this sugar down. This suggests that what little trehalose is found in some tardigrades could be derived from their food sources, rather than being made de novo by the water bears themselves [7, 20]. These observations and experiments do not imply that trehalose is not a potent mediator of anhydrobiosis, but rather suggest that it is not the only molecule that can confer desiccation tolerance. Some organisms, like tardigrades and rotifers, may rely on a completely different or augmented set of protectants.

If remarkably stress tolerant animals such as tardigrades and rotifers are not using trehalose to survive drying, what they might be using instead quickly became a pressing question in the desiccation tolerance field. While there are doubtless many molecules that contribute to anhydrobiosis, a paradigm that has emerged in the field is that many desiccation tolerant organisms accumulate small, intrinsically disordered proteins (IDPs) to immense levels when exposed to drying conditions [6]. For example, while Bdelloid rotifers do not accumulate trehalose during drying, they do amass large quantities of small, hydrophilic, intrinsically disordered proteins called Late Embryogenesis Abundant (LEA) proteins [15]. LEA proteins were first identified in cotton seeds and accumulate to high levels at a time that correlates with the embryo becoming desiccation tolerant [21]. Since their discovery in cotton seeds, LEA and LEA-like proteins have been found in myriad anhydrobiotic organisms including other plants, animals, fungi, bacteria, archaea, and protists [6, 22]. While many LEA proteins have known or suspected roles in anhydrobiosis, it should be noted that because LEA proteins were named for the time when they become abundant in seed development (late embryogenesis) rather than for a particular functional role, not all LEAs participate in desiccation tolerance. For more information on the many distinct groups of LEA proteins and their various functions, we direct the reader to the numerous reviews on the topic, such as Battaglia et al. [22] or Hincha and Thalhammer [23].

Since tardigrades are unlikely to accumulate trehalose to high levels, and Bdelloid rotifers appear to use LEA proteins rather than trehalose to survive drying, the quest to identify LEA genes in tardigrades began. While tardigrades do possess bona fide LEA genes [24, 25], the more exciting discovery was that tardigrades have several unique families of disordered proteins [24, 25], collectively known as tardigrade disordered proteins (TDPs) [7]. This review focuses on the discovery and characterization of TDPs, their properties, functions, and potential mechanisms of desiccation protection.

## Main text

The discovery of the first two families of TDPs, cytoplasmic- and secreted- abundant heat soluble (CAHS and SAHS) proteins, was made in 2012 by Yamaguchi et al. while performing heat-soluble proteomics in a search for LEA and LEA-like proteins in the tardigrade *Ramazzottius varieornatus* [25]. As highly disordered proteins, many LEA proteins have the peculiar property that they are heat-soluble, meaning that they can remain in solution even when boiled, while most other proteins denature, aggregate, and precipitate out of solution. This ability of disordered proteins to avoid precipitation when exposed to changing, often extreme conditions, is not restricted to high-temperatures, and has been used previously for the isolation and characterization of many IDPs, for example with perchloric and tricholoracetic acids [26]. IDPs generally do not aggregate and precipitate when exposed to high-temperatures or when exposed to extreme pHs since for highly disordered and charged proteins such conditions do not promote large-scale changes to structure [27]. Conversely, for well-folded proteins, denaturing temperatures and extreme pH induced change imbalances lead to myriad perturbations ranging from disruption of disulfide bridges to dissociation of protein subunits [28, 29]. A result of many of these perturbations is the formation of a random-coiled state, which for formerly well-folded proteins, unlike IDPs, contain a high proportion of aggregation prone hydrophobic residues that when exposed promote aggregation and precipitation [30, 31]. Performing heat-soluble proteomics on extracts from the tardigrade *R. varieornatus* revealed two abundantly expressed classes of proteins; CAHS and SAHS. These new protein families are distinct from LEA proteins at the level of sequence and domain conservation, and share little or no similarity at the sequence level to genes or proteins outside the phylum Tardigrada. In a later publication by Tanaka et al. [24], a third TDP family was described, the mitochondrial abundant heat-soluble (MAHS) protein family.

**Fig. 1:**
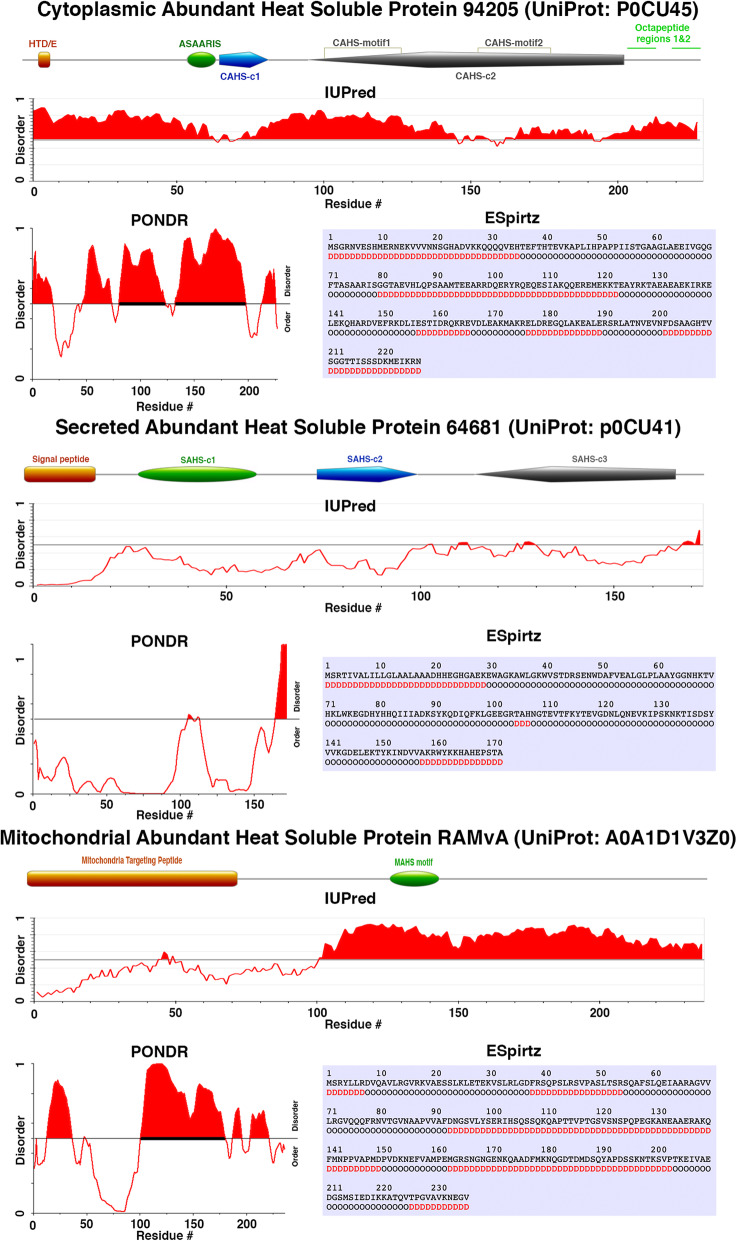
Distribution of domains and predicted conformational disordered in the three tardigrade disordered protein families. For each of the three TDP families, a scaled schematic diagram shows domains typifying proteins in the family. Below each schematic are predictions of disorder made with three different predictors: IUPred [34], PONDR [35], and ESpritz, [36] showing the disordered tendency for a representative protein from the family

TDPs appear to be unique to tardigrades, with most possessing no sequence homology to non-tardigrade genes, proteins, or conserved domains [7, 24, 25]. There are a few exceptions, such as SAHS proteins which show low sequence homology to the Fatty Acid Binding Protein (FABP) family [32, 33]. The low homology to FABPs seen for some SAHS proteins mirrors the lower predicted disorder for the SAHS family relative to other TDPs [7]. Furthermore, within TDP families there is conservation of disordered tendency and distribution, yet between TDP families the tendency and distribution of disorder is not conserved (Fig. 1) [7]. CAHS proteins are highly disordered across their entire length. MAHS proteins are predicted to have more ordered N-terminal regions (~ 100 amino acids) while ~ 150 residues in their C-terminus appear highly disordered. SAHS proteins, as mentioned above, lack strong predicted disorder. Despite these differences, all TDPs are heat-soluble [7].

While CAHS, SAHS, and MAHS proteins lack strong homology to non-tardigrade proteins and domains, there are conserved motifs that are distinct to each of these TDP families (Fig. 1). MAHS proteins share common features, such as an N-terminal mitochondrial signal peptide and an 18-mer MAHS motif, which is characterized by a predominance of hydrophilic and hydrophobic residues, and is predicted to form an amphipathic helix (Fig. 1) [24]. SAHS proteins are united by a conserved signal peptide in their N-terminus, followed by three conserved domains SAHS-c1, SAHS-c2, and SAHS-c3 (Fig. 1) [25]. All CAHS proteins analyzed to date show conservation within four regions: an N-terminal HTD/E region, a conserved ASAARIS motif, and the CAHS-c1 and CAHS-c2 regions. The CAHS-c2 region can be further broken down into two 19-mer CAHS-motifs, with each CAHS-motif being made up of two octapeptides bridged by a three amino acid linker (Fig. 1) [25]. It is of note that these conserved TDP motifs sequences are distinct from those of LEA proteins both in terms of primary sequence as well as distribution of physiochemical properties [25]. Given this, and the fact that tardigrades possess canonical LEA proteins [24], one could speculate that TDPs are not simply highly derived LEA proteins, and may possess novel functions and/or work through distinct mechanisms.

While the CAHS, MAHS, and SAHS families have conserved sequence and physiochemical properties that are distinct from that of LEA proteins, there are some similarities between TDPs and LEAs. For example, many TDP and LEA proteins are bioinformatically predicted to form helices upon dehydration [24, 25]. Furthermore, at least one SAHS and one CAHS protein have been shown, using circular dichroism spectroscopy, to form helices when exposed to increasing levels of the desolvating agent trifluoroethanol (TFE) [25], indicating that upon desiccation these TDPs may undergo structural shifts. It should be noted that sufficient levels of TFE will induce most proteins to form helices, and as discussed below LEA protein secondary structure varies depending on the chemical environment and the type of biomaterial they are protecting. Thus, the functional significance of CAHS and SAHS helical shifts still requires validation.

It is clear that much work is needed in order to resolve the functions and mechanisms by which TDPs operate during stress tolerance. Below we present possible roles and mechanisms for MAHS, SAHS, and CAHS proteins.

### The mitochondrial abundant heat soluble protein family

The MAHS family of TDPs is likely responsible for one of the most critical jobs in desiccation tolerance, protecting mitochondria from harm. Mitochondria and peroxisomes are responsible for creating as well as eliminating reactive oxygen species (ROS) during normal cellular metabolism. If unchecked ROS can cause harm throughout the cell; by damaging membrane lipids, crosslinking proteins and DNA, destroying RNA, and creating other destructive chemical species [37–40]. Tardigrade MAHS proteins are predicted to localize to mitochondria, a fact confirmed by ex vivo expression of tagged MAHS proteins in mammalian cells [24]. Constitutive expression of individual MAHS family proteins in human cells protected these cells from a loss of metabolic activity when shocked with hyperosmotic conditions, relative to cells not expressing MAHS proteins [24]. This implies that even in a different cellular context than their evolved niche, MAHS proteins are capable of preserving mitochondrial function during osmotic stress.

A possible mechanism through which tardigrades could mitigate damage induced by desiccation to mitochondria is through global changes to the mitochondrial membrane system. Since the enzymes responsible for generating ROS reside within either the mitochondrial inner membrane or the inner membrane space [41], a reduction in surface area and volume of these regions would decrease the mitochondria’s capacity to produce ROS when dried [42]. Richaud et al. [43], found that in desiccated tardigrades, mitochondria are both smaller and less membranous than in hydrated tardigrades, with a clear loss of inner membrane cristae. The loss of mitochondrial cristae and decrease in membrane surface area in dry animals relative to hydrated animals suggests that these organelles are less metabolically active, and therefore may generate less ROS as they dry. While the morphology of mitochondria is altered in dry tardigrades, there is no obvious loss of mitochondrial integrity associated with desiccation. In fact, it was found that the mitochondria of rehydrated animals are intact, and larger than in the desiccated state [43]. Since MAHS family proteins localize to mitochondria [24], they may play a role in preparing the mitochondria for desiccation through reducing the mitochondria’s ability to produce harmful ROS. This could be accomplished through the reorganizing and minimizing of mitochondrial cristae. In addition, since mitochondria in rehydrated tardigrades appear to be intact, MAHS proteins may play a role not only in membrane structural remodeling, but also in maintaining membrane integrity.

**Fig. 2:**
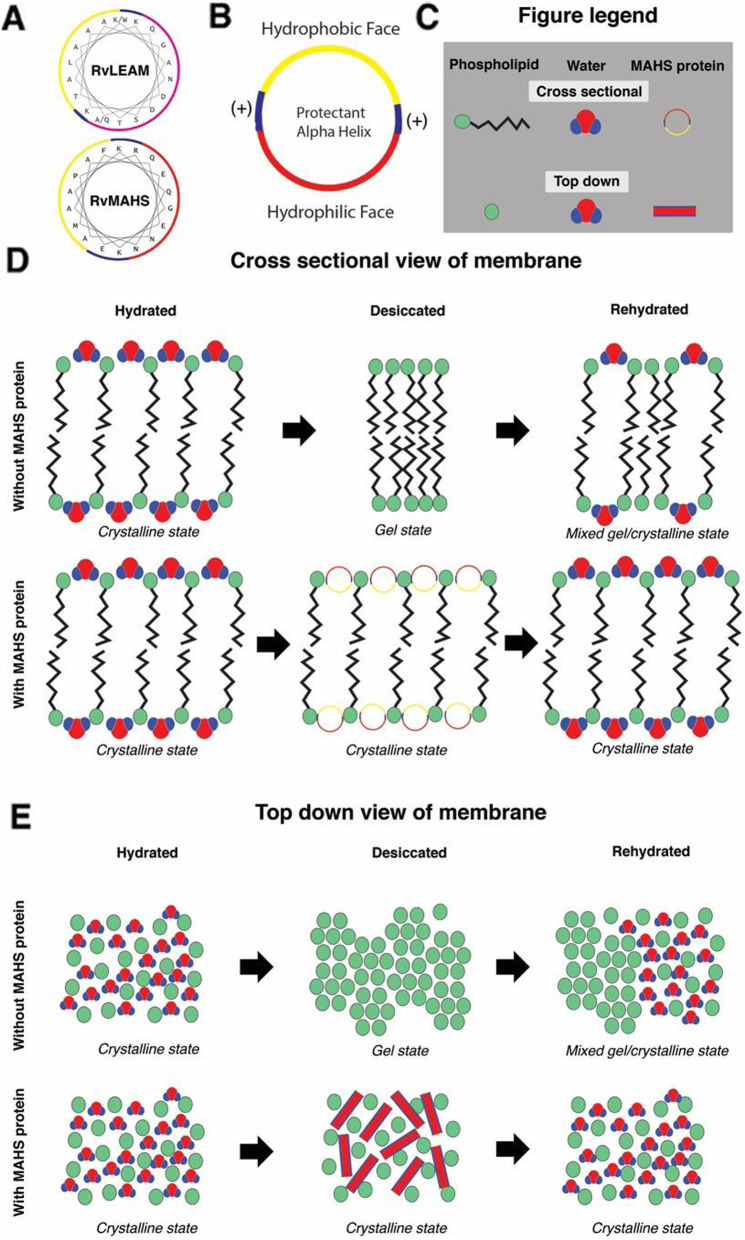
Proposed stabilization of membranes by MAHS helices. **a** Proposed helical wheel for RvMAHS and RvLEAM (adapted from [24]). **b** Schematic representation of proposed RvMAHS helix. **c** Figure legend for (**d & e**). **d** Cross sectional view of a phospholipid bilayer. Top panel shows phospholipid bilayers without protection from MAHS proteins in a hydrated (crystalline), desiccated (gel), and rehydrated (mixed gel/crystalline) states. Lower panel shows phospholipid bilayers with protection from MAHS proteins in a hydrated (crystalline), desiccated (crystalline), and rehydrated (crystalline) states. **e** Top down view of a phospholipid bilayer. Top panel shows phospholipid bilayers without protection from MAHS proteins in a hydrated (crystalline), desiccated (gel), and rehydrated (mixed gel/crystalline) states. Lower panel shows phospholipid bilayers with protection from MAHS proteins in a hydrated (crystalline), desiccated (crystalline), and rehydrated (crystalline) states

In order to understand the molecular details of maintaining membrane integrity, it is important to address what happens to a membrane as it dries. A critical aspect of retaining membrane integrity in response to osmotic stress is the ability to maintain or restore membrane fluidity. Phospholipid membranes can exist in different phases, the most relevant being the lamellar liquid crystal phase and the lamellar gel phase [44]. The liquid crystal phase is characterized by high membrane fluidity, decreased membrane thickness and less packed head groups. The gel phase is the opposite: membranes are less fluid, thicker, and have densely packed phospholipid head groups [45, 46]. In the case of desiccation, the volume change that occurs as water is lost causes membrane lipids to become closely packed into the gel phase (Fig. 2d and e), even if the membrane composition is unchanged [47]. When a desiccated membrane is rehydrated, the transition from the gel phase back to the liquid crystal phase does not occur uniformly across the membrane; some regions remain packed in the gel phase while others spread out in the liquid crystal phase (Fig. 2d and e) [48–50] . Because of this non-uniform phase transition the membrane cannot expand to accommodate increased water volume during rehydration, ultimately causing transient holes to form in the membrane. In order to preserve membrane integrity, appropriate spacing between the polar head groups of membrane phospholipids must be maintained. The precise thickness of a membrane and spacing needed between phospholipid headgroups is a function of the membrane’s composition, which may explain the presence of many putative membrane-protective IDPs in the LEA and TDP families.

One mechanism through which MAHS family proteins may protect membranes is through preventing the transition from the liquid crystal to the gel state by maintaining proper phospholipid head group spacing (Fig. 2d and e). Several LEA proteins suspected to mediate membrane protection during desiccation are thought to maintain the spacing between polar head groups, preventing the membrane from transitioning into the gel phase [51–54]. For example, group 3 LEA proteins that preserve membrane integrity have been shown to undergo a structural transition upon desiccation to α-helices. These LEA helices have a particular arrangement of charged and uncharged residues in the helix [51–54], which is speculated to allow them to intercalate between phospholipid head groups, thus maintaining proper head group spacing and preventing gel formation (Fig. 2a, d and e). The mitochondrial LEA protein *Ps*LEAM [54] folds in such a way that the α-helix that is formed has negatively charged residues collected in a longitudinal stripe along the bottom face of the helix, with non-polar residues oriented in a stripe directly opposite the negative charges along the top face of the helix (Fig. 2a and b). Sandwiched between these stripes, on both sides of the helix, are positively charged residues (Fig. 2b). This configuration allows the non-polar residues of the protectant-helix to bury into the membrane just past the phospholipid head groups, protruding into the region of the membrane populated by lipid chains (Fig. 2d). The negatively charged face of the helix is thus oriented into the cytoplasm, while the positively charged stripes are positioned between the negatively charged phospholipid head groups of adjacent membrane phospholipids (Fig. 2d and e) [54]. MAHS family proteins show similar arrangements of charged and uncharged residues [24] (Fig. 2a), indicating they may follow a similar paradigm of helical formation that could allow for crucial membrane associations and stabilization of membranes in a liquid crystalline state.

While adoption of helical structure is often ascribed to LEA and other stress tolerant IDPs, the context and environment that a disordered protein is in must be taken into account when considering its function. Due to their increased surface area and malleable nature, disordered proteins are much more sensitive to their environment than their folded counterparts [55–58]. An example of this is the peptide *Pv*LEA-22, which is composed of two repeats of the consensus sequence of the group 3 LEA protein 11-mer motif found in the midge *Polypedilum vanderplanki*. This peptide has an extended, unfolded chain conformation when protecting proteins from aggregation [59, 60], adopts β-sheet structure in the presence of membranes [61], and an α-helical structure when dried in isolation [62]. While these observations demonstrate the malleability of IDPs and the influence of the environment on their conformational ensemble, they also serve to highlight the potential for a single stress-tolerant IDP to mediate many protective functions in the cell. While MAHS proteins are predicted to form helices, how intracellular conditions and interaction with client molecules influence this propensity remains to be seen. It is possible that, like the *Pv*LEA-22 peptide, MAHS proteins may have different dynamic and functional properties depending on the environment in which they are studied.

### The secreted abundant heat soluble protein family

As outlined above, most of the speculation on the function of MAHS proteins is based on secondary structural predictions, and the similarities of these predictions to better-characterized LEA proteins. Unlike MAHS proteins, SAHS proteins have regions of much lower predicted disorder, which makes these domains of SAHS proteins amenable to crystallization [7, 25]. Crystallographic studies have allowed researchers to gain structure-based insights into the possible functions of SAHS proteins that are not possible for either the MAHS or CAHS proteins.

A series of crystallographic studies on the SAHS proteins in *Ramazzottius varieornatus* have revealed the similarity between some SAHS proteins and a versatile class of proteins called fatty acid binding proteins (FABPs). The structures of *Rv*SAHS1 [33] and *Rv*SAHS4 [32] are similar to other canonical FABPs, characterized by an antiparallel β-barrel with an internal FA binding pocket, and a helix-turn-helix lid. In their 2017 study, Fukuda et al. found that *Rv*SAHS1 binds to small fatty acids, but that bulky residues present in its two ligand binding sites (LBS1 and LBS2) prevent this protein from binding large membrane phospholipids. In their later 2018 study, Fukuda and Inoue found that both LBS of *Rv*SAHS4 are even more occluded than *Rv*SAHS1. *Rv*SAHS4 was only able to accommodate a small molecule of PEG in its LBS2, while the LBS1 was completely blocked by the lid of the β-barrel.

In addition to the different sizes of LBS in the two SAHS paralogs, there were also important differences in the polarity of amino acids within the LBS of *Rv*SAHS1 and *Rv*SAHS4. While some polar amino acids are conserved, other critical residues needed to bind to negatively charged FAs are replaced by neutral side chains in *Rv*SAHS4. Moreover, sequence analysis of 13 SAHS proteins from *R. varieornatus* and 6 SAHS proteins from *H. exemplaris* show that while there is conservation of the ligand binding site, the polarity of amino acids in positions relevant for ligand binding did differ in many of the SAHS family homologs. These differences in LBS size and charge imply diversity in binding capability and specificity within the SAHS family of proteins. Given that FABPs are capable of bindings a wide range of molecules beyond just fatty acids [63–65], the search for possible binding partners for a SAHS protein should not be limited to fatty acids, as such a partner could be any number of molecules present in the secretory pathway or extracellular space.

In addition to considering the FABP-like homology of SAHS proteins, the fact that SAHS proteins are secreted and found mostly in the media when expressed in cultured cells may hint at possible protective functions. A recent whole-organism investigation of tardigrades by Richaud et al. (2020), found an increase in the abundance of secretory cells in dried tardigrades. Associated with these dry secretory cells was a novel extracellular structure, located just outside the cell membranes, termed the special extracellular structure (SES). In dried tardigrades, SES form around secretory cells, producing an encompassing structure superficially reminiscent of a plant cell wall. Partially rehydrated tardigrades had fewer secretory cells and an intermediate level of SES, which were overall less well-structured than those of dry tardigrades. Once the animals were fully hydrated, no secretory cells were found in the five animals examined, and SES were absent. Since SAHS proteins are secreted [25], highly abundant [7, 25], and known in some cases to be essential for robust desiccation tolerance [7], it is tempting to speculate that they may be produced by secretory cells and contribute to the formation of the SES observed in dry tardigrades.

While a functional role for the SES in mediating desiccation tolerance has not been established, its appearance only during drying implies some role(s) in preventing damage induced by drying. The SES could protect tardigrades and their cells from a number of deleterious desiccation-induced perturbations. The SES and its constituents could serve as a means by which to stabilize membranes and prevent gel-crystalline phase transitions that result in leakage, as discussed for MAHS proteins. This structure may serve to separate the plasma membranes of adjacent cells, preventing their fusion. The SES may also provide structural support to cells as the organism enters a desiccated state, preventing a complete collapse and loss of macro-scale structure.

When considering whether SAHS could be involved in the SES, it is important to remember that the crystal structures of *Rv*SAHS1 and *Rv*SAHS4 were derived from truncated SAHS proteins, which lacked their disordered regions. The FABP-like regions of SAHS proteins might interact with extracellular components of the plasma membrane, while the disordered regions could interact with one another and/or undergo a structural transition when dried, similar to that seen in LEA proteins. These restructured SAHS proteins might make quaternary contacts, like the coiled-coil of α-helices described in modeling experiments by Nishimoto et al. [66]. Ultimately such multi-protein structures could form the thick SES seen in dried tardigrades. Even if there is not a structural transition in the dry state, simply including the disordered regions of SAHS proteins could allow for some degree of plasticity in structure and function. Perhaps the disordered regions expand the client binding capabilities of SAHS proteins by enlarging the binding surface, or allowing for a structural shift to accommodate a client.

### Cytoplasmic abundant heat soluble proteins

Functional experiments on CAHS proteins have demonstrated the vital role of this family in desiccation tolerance. Many CAHS family proteins are expressed at high levels, are upregulated in response to drying, or are required for tardigrades to robustly survive desiccation. CAHS proteins have also been shown to prevent desiccation-induced protein damage in vitro, and improve desiccation tolerance in heterologous systems such as yeast and bacteria [7]. Clearly these proteins are an integral part of the tardigrade response to desiccation, yet the mechanism(s) by which these proteins function are not well defined. There are several theories in the field of anhydrobiosis regarding how desiccation tolerance may be mechanistically mediated, through which CAHS proteins may function: these are the vitrification, water replacement, and preferential hydration hypotheses. These theories, as well as the known and potential links of CAHS proteins to them, are discussed below. It should be noted that these hypotheses are not mutually exclusive, so there is the potential for CAHS proteins to function through several, or all, of these mechanisms.

A longstanding theory in the field of desiccation tolerance is the vitrification hypothesis [67, 68]. The vitrification hypothesis posits that as an organism dries, the intracellular viscosity could be increased to such a degree that the detrimental effects of desiccation, such as protein denaturation and membrane fusion, would slow to a rate at which they essentially stop [68]. A key aspect of this theory is that the increase in viscosity be mediated by an accumulation of molecules that form vitrified or amorphous solids, rather than crystalline solids, when dried. In a biological setting, crystals can induce a number of deleterious effects. Traditionally, the vitrification hypothesis has been associated with the disaccharide trehalose [12, 68]. As detailed above, trehalose is a well-established functional mediator of desiccation tolerance that accumulates to high levels in many anhydrobiotic systems, and is known to undergo vitrification upon dying [12, 68]. While vitrification in many organisms correlates with desiccation tolerance [7, 12, 63], it does not appear to be sufficient to mediate anhydrobiosis in all cases [62], suggesting that other essential mechanisms likely exist.

In their seminal study, Hengherr et al. [69] used differential scanning calorimetry to establish that tardigrades vitrify upon desiccation, and that their vitrified state correlates with their survival, suggesting a link between vitrification and viability [69]. As mentioned above, the role of trehalose in mediating tardigrade desiccation tolerance is unclear, with some species of tardigrade accumulating low levels of the sugar, while other species have no detectable trehalose [17–19]. Furthermore, genomic and transcriptomic sequencing suggests that tardigrades may lack the genes required to make trehalose [7, 20]. The low levels, or complete lack, of trehalose in dry tardigrades suggests that other mediators of desiccation tolerance may play a role in the vitrification of these animals. Many tardigrade species require relatively slow rates of initial drying to survive complete desiccation [7, 70], a phenomenon that could be associated with the need to sense that they are drying and produce sufficient levels of protectants to survive this insult [7]. It was recently found that slowly dried tardigrades, which have time to accumulate protectants and survive drying, are vitrified when dried [7, 69]. Conversely, tardigrades that are dried quickly and cannot accumulate protectants do not vitrify, and do not survive desiccation [7]. CAHS proteins are obvious candidates for mediating vitrification in tardigrades because they are both disordered, implying that they cannot crystalize, and are some of the most abundantly expressed proteins during desiccation [7]. CRISPR, or other genome editing techniques, have yet to be developed in tardigrades, making direct tests of the vitrifying properties of CAHS proteins in these animals impossible. However, in vitro experiments on purified CAHS proteins indicate that they do vitrify when dried [7]. Furthermore, expression of CAHS proteins in yeast result in the formation of a novel vitrified material upon desiccation, with CAHS-expressing yeast survival correlating with the maintenance of this vitrified state [7]. Thus, given their ability to vitrify in vitro and in heterologous systems, as well as their abundance in desiccating tardigrades, CAHS proteins may play a role in the ability of tardigrades to vitrify upon desiccation.

The glass transition temperature (T_g_), defined as the temperature at which a vitrified solid transitions to a rubbery solid, differs between dried whole tardigrades (~ 100 °C), purified CAHS proteins desiccated in vitro (~ 60 °C & ~ 135 °C), and desiccated yeast expressing CAHS proteins (~ 75 °C) [7]. At first glance, this fact may seem to imply that CAHS proteins are not mediating vitrification in these different settings, since the T_g_ varies between these different contexts. However, since the environment in which a vitrified solid is formed has a large influence on its physical properties [71, 72], it is expected that in vitro CAHS proteins would have a different T_g_ than CAHS proteins in a yeast cell or in a tardigrade. Furthermore, IDPs are more readily influenced by their chemical environment than their folded counter parts, due to their increased surface area and reduced intraprotein interactions [55–58]. Along these lines, it has been observed that the ambient humidity in which dried CAHS proteins are stored modulates their Tg, with increasing humidity weakening the vitrified state (Boothby, Unpublished). Additionally, incubation of co-solutes with CAHS and other IDPs results in mixtures that form vitrified solids with dramatically different thermal properties (Boothby, Unpublished). Thus, while CAHS proteins appear linked to vitrification in tardigrades, and tardigrade vitrification correlates with survival in a dried state, it is likely that other molecules and co-solutes modulate the physical, thermal, and functional properties of vitrified CAHS proteins.

It should be noted that in a desiccated state, tardigrades and other anhydrobiotic organisms still retain some water, but this water is likely insufficient to fully hydrate their full proteome and other biomolecules [73–75]. The coordination of water in a vitrified solid is crucial to the stability of the solid state [76, 77], yet precisely how this water coordination occurs is still debated. There are several prominent hypotheses that address how desiccation protectants compensate for changes in the hydrogen bonding network (HBN) between water and a solvated protein in a desiccated system.

The water replacement hypothesis posits that the protectant itself, for example trehalose or glycerol, directly replaces the HBN between water and the protein [78–80], thus the stabilizing hydrogen bonds usually made with water are replaced by hydrogen bonding with the protectant. The ability to replace bonds typically made by water is attributed to the polar nature of the protectant, which gives it the ability to form an HBN directly with the protein’s hydrophilic surface [68, 79, 81, 82].

An alternative model, the preferential hydration hypothesis, states that a mediator of desiccation tolerance might function by preserving a hydrating layer of water on the hydrophilic surface of the protein. There have been two proposed means through which preferential hydration could occur, water entrapment or preferential exclusion. Water entrapment involves trapping a layer of water between the protectant and a desiccation-sensitive protein. Preferential exclusion entails crowding water molecules away from the protectant, thereby pushing water to form more contacts with the protein surface. Water entrapment seems to require a lack of intramolecular hydrogen bonding within the protectant molecule [82, 83], while preferential exclusion involves extensive intermolecular bonding on the part of the protectant with other protectant molecules [77, 84]. One might expect such mechanisms to be mutually exclusive, yet there is experimental support for trehalose using both of these mechanisms. This is reminiscent of our discussion of the contextual dependence of IDP conformations, as it implies that the mechanisms through which trehalose provides desiccation protection may vary depending on the conditions of the protein system being studied. Indeed, it has been found that temperature, protein character, protein:sugar molar ratios, and the degree of dehydration, can all impact how trehalose and water behave [77, 80] with some studies suggesting that both mechanisms of preferential hydration could occur simultaneously within a particular system [77, 79].

Both the water replacement and preferential hydration hypotheses require that a protectant be hydrophilic, with the ability to order water, without an extensive internal hydrogen bonding network [81, 83]. CAHS proteins have the ability to protect desiccation-sensitive proteins [7, 8], and members of this family fall in line with the characteristics needed to conform to these two solvation-based hypotheses [25]. CAHS proteins are larger than trehalose and other disaccharides, and therefore capable of crowding water to the surface of the client protein. Another important factor to consider when assessing the validity of applying solvation-based mechanisms to CAHS family members is the distribution of charged amino acids within these proteins, and whether these proteins can supplant the HBN of water or coordinate a hydration layer. In order to achieve these hydrogen bonding requirements, CAHS proteins should have a polyampholytic character. Polyampholytes, as defined by Das et al. [85], are proteins with roughly equivalent numbers of positive and negative charges distributed evenly through the sequence. The CAHS motif and the octapeptide motif, each found twice in CAHS family protein sequences, have well distributed charges [25] which would likely give the protein an overall polyampholytic character.

**Fig. 3:**
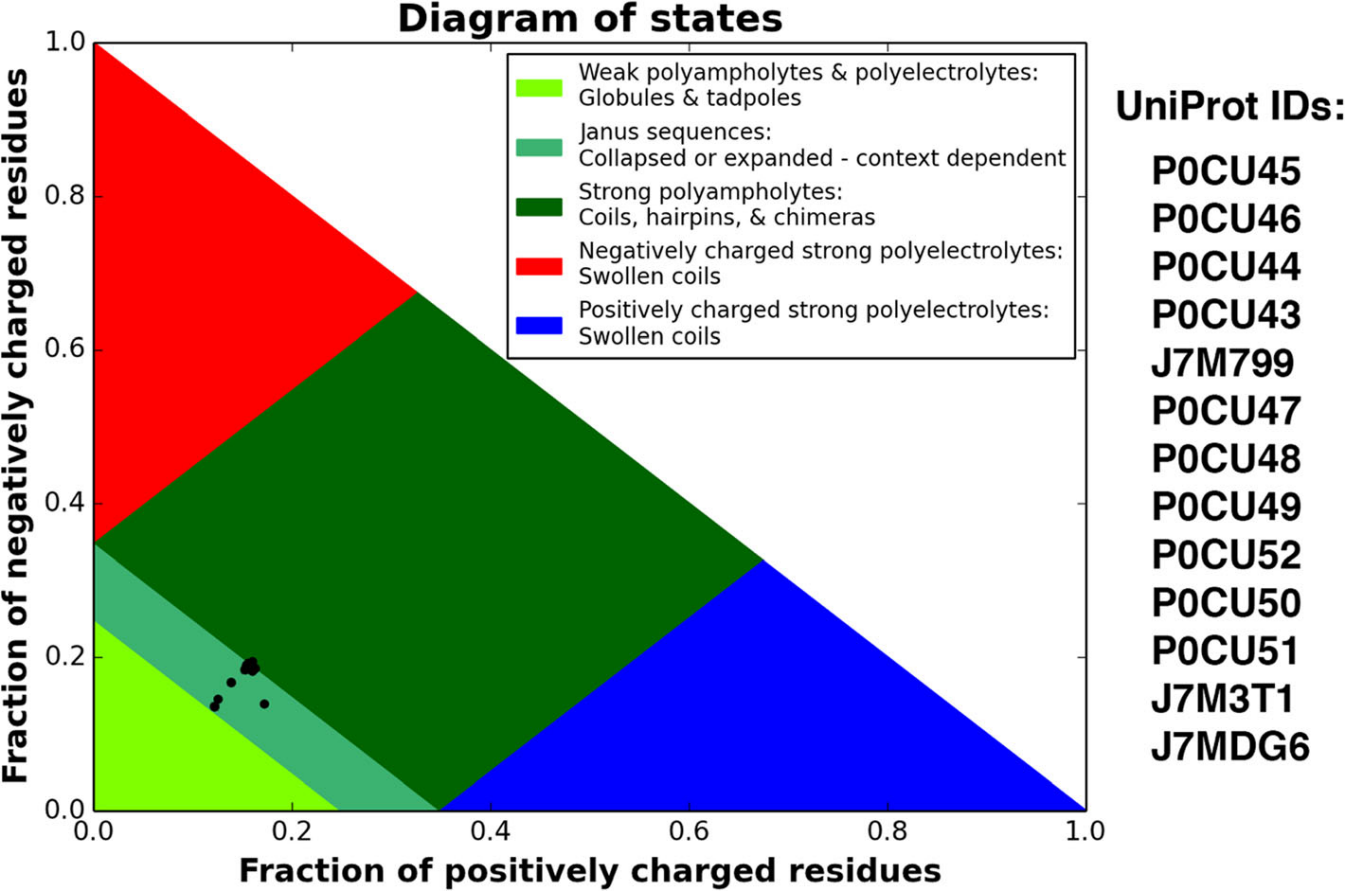
Das plot showing the fraction of positively and negative charges residues for all CAHS proteins deposited in UniProt

Das et al. [85, 86] and Holehouse et al. [87] constructed a framework for understanding and predicting the conformational dynamics of intrinsically disordered proteins from primary sequence information. Das et al. [85] show that the fraction of charged residues, and the distribution pattern of those residues in the primary sequence, can dictate the conformational space of a polyampholytic IDP. Construction of a Das plot for CAHS proteins (Fig. 3) suggests that members of this protein family exist as polyampholytes; proteins that are able to move rapidly between elongated conformations of stochastically formed coils and more collapsed forms [85, 87, 88]. It is important to note that the Das plot of CAHS proteins (Fig. 3) shows a spectrum of putative characteristics. Some CAHS proteins firmly occupy the “Janus” region of the plot, while other members fall more towards the “Strong polyampholyte” region, and still others towards the “Weak polyampholyte” region. Those CAHS proteins trending towards strong polyampholyte group are more likely to maintain their elongated state, which would make those particular proteins better able to coordinate water. CAHS D (UniProt: P0CU50), which was found by Boothby et al. [7] to form vitrified solids and increase the survival of desiccated yeast and bacteria, is among the CAHS proteins clustered towards the strong polyampholyte region of the Das plot (Fig. 3). The elongated state of strong polyampholytes, along with the hydrophilic nature of the CAHS sequences and the alternation of positive, negative and polar residues, provides the perfect scenario for coordinating water. There is ample opportunity for hydrogen bonding between water and the extended protein chain, and the lack of rigid secondary structure means that even backbone residues could add to the hydrogen bonding network. Therefore, from a structural standpoint, some CAHS proteins have the potential to be potent coordinators of water networks. The range of characteristics seen in the Das plot of CAHS proteins implies that there is diversity in function among the various CAHS family members, and which could be uniquely tuned for protecting biomolecules in different cellular contexts. Overall the diverse nature of the CAHS family belies an underlying theme for protective IDPs; that multiple functions and mechanisms of protection can be performed by a single protein.

## Future perspectives

Beyond fully defining the functional and mechanistic underpinnings of TDP-mediated stress tolerance, many fascinating questions addressing the biology of these proteins remain to be answered. One question of immense interest is where TDPs originated. Are TDPs just highly derived LEA proteins? If so, did their divergence in sequence from LEA proteins result in novel functions, or were existing sequence features and functions simply fine-tuned to the specific biology of tardigrades?

In light of these questions, it is of interest that while tardigrades have evolved these three unique stress-related TDP families, they also possess bona fide LEA genes [24]. It has sometimes been erroneously assumed that all LEA proteins function similarly, though this is clearly not the case, especially between different LEA families. It would therefore be imprudent to assume that TDPs function similarly to LEA proteins, or that all TDPs function similarly to each other. Certainly, some similarities between TDPs and LEAs appear to exist. For example, many LEA proteins form α-helices when desiccated and many TDPs are predicted to do the same [24, 25]. However, the functional significance of adopting α-helical content has not been demonstrated for TDPs, and with some LEA proteins β-sheets are the predominant secondary structure acquired when desiccated in the presence of the biological materials that they protect [61].

**Fig. 4:**
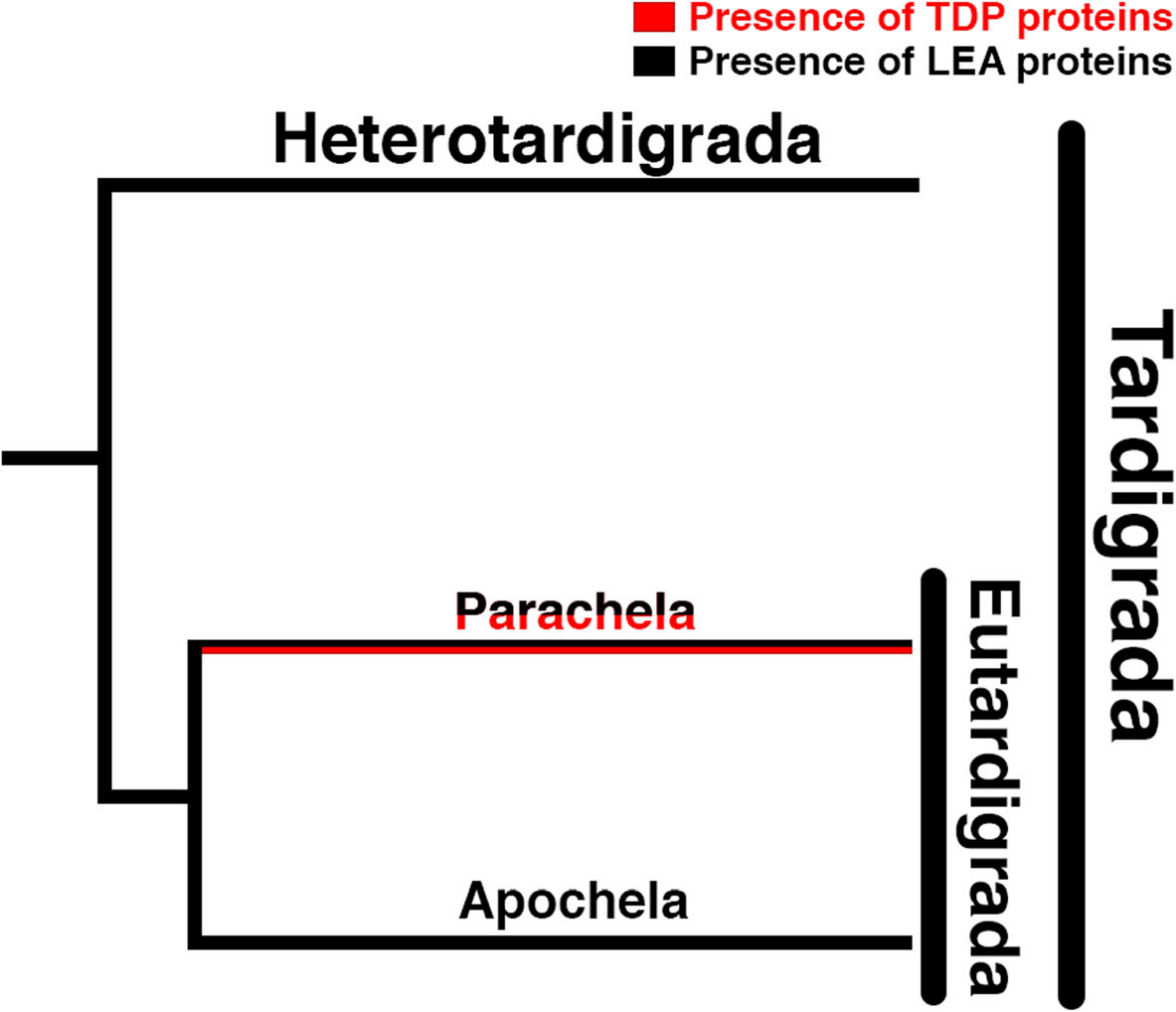
Distribution of stress tolerant IDP families among major tardigrade lineages. Red and black coloring of branches denotes the presence TDP or LEA proteins, respectively, within a clade

Work by Kamilari et al.*,* compared gene families found in species from both of the two main tardigrade lineages, eu- and heterotardigrades [89]. Transcriptomic analysis of heterotardigrades did not reveal the presence of any canonical CAHS, SAHS, or MAHS proteins, while many eutardigrade species possess these TDP families. This suggests that TDPs likely arose during or after the eutardigrade/heterotardigrade split (Fig. 4). Further investigation of EST libraries by Kamilari et al. [89]*,* failed to detect TDPs in an apochelan eutardigrade, yet all members of the eutardigrade order Parachela, assessed to date, possess TDPs. While genome-wide analysis of TDP content in additional species would bolster these initial reports derived from transcriptomic and EST datasets, they are nonetheless intriguing. Indeed, these studies have provided striking evidence that different tardigrade species likely use different means to cope with environmental stresses, and while TDPs are important for some species, they may be dispensable or completely absent in others.

Several additional points of interest arise from the Kamilari study. First, while TDPs were not identified in heterotardigrade and apochelan species, LEA proteins were identified in all tardigrade species (Fig. 4). Whole transcriptome comparison of the heterotardigrade *E. sigismundi* with eutardigrade, animal, and fungal species reveals a large divergence at the level of sequence conservation between hetero- and eutardigrades. Since IDPs are known to diverge more quickly than their structured counterparts [90, 91], it is possible that TDPs could have arisen earlier in the tardigrade lineage, and their signal was subsequently lost due to rapid sequence evolution in different lineages. Finally, it is also interesting to note that the three TDP families are either all present or all absent in the different species investigated, and there have been no species found with piecemeal assemblages of these families. This implies that these distinct protein families may work synergistically, or, in light of their distinct subcellular localization, may replace the function of a different protectant that would typically have a more widespread cellular distribution.

Another pressing question is how the environment influences the conformational and functional properties of a TDP. Due to their increased surface area and relatively low occurance of intramolecular bonds, IDPs are known to be influenced by their environment to a larger degree than globular proteins [55–58]. In some cases, changes in the environment of an IDP could lead to it becoming non-functional, but in other circumstances this responsiveness to the environment may provide an elegant way for an IDP to gain or augment functionality. In this regard, TDPs may provide an interesting evolutionary example of cross-tolerance; when a mediator that has evolved to cope with one stress confers protection to a different stress. CAHS proteins, for example, have been seen to function in vitro to protect proteins from both desiccation and freeze/thaw induced perturbations [7, 8]. While freezing, like desiccation, removes solvating water, transcriptome level responses to these two stresses are highly divergent [7]. The complement of small molecules and co-solutes that are enriched or depleted in these two conditions are almost certainly profoundly different, and thus the cellular environment in whichCAHS proteins exist during these two different stresses would also vary. What influence these different environments have on CAHS proteins, and to what degree they influence CAHS structure and function, is unknown. However, there is precedence in the IDP stress tolerance field for modulation of structural and functional dynamics. As discussed previously, the 22-mer LEA4 motif of *P. vanderplanki* adopts an α-helical conformation when dried alone or in the presence of trehalose, but adopts β-sheet character when dried in the presence of membranes. Given the breadth of environmental extremes that tardigrades can survive, the question of how the changing cellular microenvironment influences TDP behavior promises to provide insights into the mechanisms underlying TDP-mediated cross tolerance.

One final question surrounding TDPs is not about their fundamental biology, but whether they might be useful in real world applications. One such application includes using TDPs to engineer stress tolerant crops, which would then be better able to cope with the Earth’s changing and increasingly extreme climate. Such technology or strategies might also be applied to model organisms, such as zebra fish or *Drosophila*, which currently lack time- and cost-efficient methods for long-term storage. A final real-world application that has been suggested is the use of TDPs as stabilizers for biomedical material, such as vaccines and other biologic pharmaceuticals, cells, or even tissues such as whole blood. Xeroprotection has been a long- standing goal of the anhydrobiosis community. Efficient and effective xeroprotective technology would relieve our dependence on the ‘cold-chain’; a series of refrigerators and freezers needed to keep many biomedical materials in a constantly cold state during manufacturing, shipment, and storage. The cold-chain represents an enormous logistical and economic hurdle for global public health, especially in remote and developing parts of the world. Learning more about the function and mechanism of TDPs may provide avenues for developing and refining xeroprotection technologies.

## Conclusions

Tardigrade disordered proteins are a diverse group of proteins that play a crucial role in anhydrobiosis, yet their precise functions and mechanisms of action are largely unknown. Like LEA proteins, the disordered nature of TDPs could allow them to sense and respond to extremely local intracellular conditions, allowing them to modulate their structural and functional properties. While the diversity of stress conditions that tardigrades tolerate may seem dissimilar, on a cellular level they can have surprising overlap. Freezing removes hydrating water from proteins and membranes analogously to desiccation, while heat stress changes the energy dynamics of a protein and destabilizes folding, akin to water loss. Irradiation induces massive damage to the genome, similarly to drying. The evolution of IDPs for stress tolerance is both an elegant and intuitive solution to address these similar-yet-different pressures. Proteins that are attuned to, and functional in, adverse cellular environments would not be ordered in normal physiological conditions. Disorder gives TDPs a broader sensitivity to their cellular environment, as they are constantly moving through different conformations, exposing different residues to the cellular microenvironment. Using disorder as a mechanism to develop stress tolerance may have allowed tardigrades to build TDPs into protein-based swiss-army knives; one protein, many functions.

Continued study of TDPs will not only help to reveal how tardigrades and other organisms protect themselves from abiotic stresses, but will also inform us more broadly about the relationship between IDP sequence and function. Additionally, the specific mechanisms of anhydrobiosis can be studied in a systematic way through mutational and physiochemical analysis of TDPs, which would provide an excellent model for studying how IDPs respond to diverse and dynamic environments.

## Data Availability

Not applicable.

## Abbreviations

IDP: Intrinsically disordered protein
TDP: Tardigrade disordered protein
CAHS: Cytoplasmic abundant heat soluble
SAHS: Secreted abundant heat soluble
MAHS: Mitochondrial abundant heat soluble
LEA: Late embryogenesis abundant
FABP: Fatty acid binding protein
FA: Fatty acid
LBS: Ligand binding site
TFE: Trifluoroethanol
HBN: Hydrogen bonding network
EST: Expressed sequence tag

## References

[1] van Leeuwenhoek A (1939). Alle de brieven.

[2] Alpert P (2006). Constraints of tolerance: why are desiccation-tolerant organisms so small or rare?. J Exp Biol.

[3] Kinchin IM (2014). The biology of Tardigrades.

[4] Jönsson KI, Rabbow E, Schill RO, Harms-Ringdahl M, Rettberg P (2008). Tardigrades survive exposure to space in low earth orbit. Curr Biol.

[5] Saragusty J, Loi P (2019). Exploring dry storage as an alternative biobanking strategy inspired by nature. Theriogenology.

[6] Boothby TC, Pielak GJ (2017). Intrinsically disordered proteins and desiccation tolerance: elucidating functional and mechanistic underpinnings of Anhydrobiosis. BioEssays.

[7] Boothby TC, Tapia H, Brozena AH, Piszkiewicz S, Smith AE, Giovannini I, Rebecchi L, Pielak GJ, Koshland D, Goldstein B (2017). Tardigrades use intrinsically disordered proteins to survive desiccation. Mol Cell.

[8] Piszkiewicz S, Gunn KH, Warmuth O, Propst A, Mehta A, Nguyen KH, Kuhlman E, Guseman AJ, Stadmiller SS, Boothby TC (2019). Protecting activity of desiccated enzymes. Protein Sci.

[9] Leslie SB, Israeli E, Lighthart B, Crowe JH, Crowe LM (1995). Trehalose and sucrose protect both membranes and proteins in intact bacteria during drying. Appl Environ Microbiol.

[10] Clegg JE, Evans D (1961). The physiology of blood Trehalose and its function during flight in the blowfly. J Exp Biol.

[11] Tapia H, Young L, Fox D, Bertozzi CR, Koshland D (2015). Increasing intracellular trehalose is sufficient to confer desiccation tolerance to Saccharomyces cerevisiae. Proc Natl Acad Sci.

[12] Sakurai M, Furuki T, Akao K-I, Tanaka D, Nakahara Y, Kikawada T, Watanabe M, Okuda T (2008). Vitrification is essential for anhydrobiosis in an African chironomid, Polypedilum vanderplanki. Proc Natl Acad Sci.

[13] Erkut C, Penkov S, Khesbak H, Vorkel D, Verbavatz J-M, Fahmy K (2011). Teymuras: Trehalose renders the Dauer larva of Caenorhabditis elegans resistant to extreme desiccation. Curr Biol.

[14] Lapinski J, Tunnacliffe A (2003). Anhydrobiosis without trehalose in bdelloid rotifers. FEBS Lett.

[15] Tunnacliffe A, Lapinski J, McGee B (2005). A putative LEA protein, but no Trehalose, is present in Anhydrobiotic Bdelloid rotifers. Hydrobiologia.

[16] Caprioli M, Krabbe Katholm A, Melone G, Ramløv H, Ricci C, Santo N (2004). Trehalose in desiccated rotifers: a comparison between a bdelloid and a monogonont species. Comp Biochem Physiol A Mol Integr Physiol.

[17] Hengherr S, Heyer AG, Köhler H-R, Schill RO (2008). Trehalose and anhydrobiosis in tardigrades - evidence for divergence in responses to dehydration. FEBS J.

[18] Ingemar Jönsson K, Persson O (2010). Trehalose in three species of desiccation tolerant tardigrades. Open Zool J.

[19] Westh P, Ramløv H (1991). Trehalose accumulation in the tardigrade Adorybiotus coronifer during anhydrobiosis. J Exp Zool.

[20] Boothby TC, Tenlen JR, Smith FW, Wang JR, Patanella KA, Osborne Nishimura E, Tintori SC, Li Q, Jones CD, Yandell M (2015). Evidence for extensive horizontal gene transfer from the draft genome of a tardigrade. Proc Natl Acad Sci.

[21] Dure L, Greenway SC, Galau GA (1981). Developmental biochemistry of cottonseed embryogenesis and germination: changing messenger ribonucleic acid populations as shown by reciprocal heterologous complementary deoxyribonucleic acid-messenger ribonucleic acid hybridization. Biochemistry.

[22] Battaglia M, Olvera-Carrillo Y, Garciarrubio A, Campos F, Covarrubias AA (2008). The enigmatic LEA proteins and other Hydrophilins. Plant Physiol.

[23] Hincha D, Thalhammer A (2012). LEA proteins: IDPs with versatile functions in cellular dehydration tolerance: figure 1. Biochem Soc Trans.

[24] Tanaka S, Tanaka J, Miwa Y, Horikawa DD, Katayama T, Arakawa K, Toyoda A, Kubo T, Kunieda T (2015). Novel mitochondria-targeted heat-soluble proteins identified in the Anhydrobiotic Tardigrade improve osmotic tolerance of human cells. PLoS One.

[25] Yamaguchi A, Tanaka S, Yamaguchi S, Kuwahara H, Takamura C, Imajoh-Ohmi S, Horikawa DD, Toyoda A, Katayama T, Arakawa K (2012). Two novel heat-soluble protein families abundantly expressed in an Anhydrobiotic Tardigrade. PLoS One.

[26] Cortese MS, Baird JP, Uversky VN, Dunker AK (2005). Uncovering the unfoldome: enriching cell extracts for unstructured proteins by acid treatment. J Proteome Res.

[27] Uversky VN (2003). A protein-chameleon: conformational plasticity of α-Synuclein, a disordered protein involved in neurodegenerative disorders. J Biomol Struct Dyn.

[28] Neumann S, Matthey U, Kaim G, Dimroth P (1998). Purification and properties of the F1Fo ATPase of Ilyobacter tartaricus, a sodium ion pump. J Bacteriol.

[29] Dill KA, Shortle D (1991). Denatured states of proteins. Annu Rev Biochem.

[30] Fink AL, Calciano LJ, Goto Y, Kurotsu T, Palleros DR (1994). Classification of acid denaturation of proteins: intermediates and unfolded states. Biochemistry.

[31] Goto Y, Calciano LJ, Fink AL (1990). Acid-induced folding of proteins. Proc Natl Acad Sci.

[32] Fukuda Y, Inoue T (2018). Crystal structure of secretory abundant heat soluble protein 4 from one of the toughest “water bears” micro-animals Ramazzottius Varieornatus. Protein Sci.

[33] Fukuda Y, Miura Y, Mizohata E, Inoue T (2017). Structural insights into a secretory abundant heat-soluble protein from an anhydrobiotic tardigrade, *Ramazzottius varieornatus*. FEBS Lett.

[34] Mészáros B, Erdős G, Dosztányi Z (2018). IUPred2A: context-dependent prediction of protein disorder as a function of redox state and protein binding. Nucleic Acids Res.

[35] Xue B, Dunbrack RL, Williams RW, Dunker AK, Uversky VN (2010). PONDR-FIT: a meta-predictor of intrinsically disordered amino acids. Biochim Biophys Acta, Proteins Proteomics.

[36] Walsh I, Martin AJM, Di Domenico T, Tosatto SCE (2012). ESpritz: accurate and fast prediction of protein disorder. Bioinformatics.

[37] Chandra J, Keshavkant S (2018). Desiccation-induced ROS accumulation and lipid catabolism in recalcitrant Madhuca latifolia seeds. Physiol Mol Biol Plants.

[38] Laxa M, Liebthal M, Telman W, Chibani K, Dietz K-J (2019). The role of the plant antioxidant system in drought tolerance. Antioxidants.

[39] Kranner I, Birtić S (2005). A modulating role for antioxidants in desiccation tolerance. Integr Comp Biol.

[40] Cadet J, Wagner JR (2013). DNA base damage by reactive oxygen species, oxidizing agents, and UV radiation. Cold Spring Harb Perspect Biol.

[41] Wollweber F, Von Der Malsburg K, Van Der Laan M (2017). Mitochondrial contact site and cristae organizing system: a central player in membrane shaping and crosstalk. Biochim Biophys Acta, Mol Cell Res.

[42] Cogliati S, Frezza C, Soriano M, Varanita T, Quintana-Cabrera R, Corrado M, Cipolat S, Costa V, Casarin A, Ligia (2013). Mitochondrial cristae shape determines respiratory chain supercomplexes assembly and respiratory efficiency. Cell.

[43] Richaud M, Le Goff E, Cazevielle C, Ono F, Mori Y, Saini NL, Cuq P, Baghdiguian S, Godefroy N, Galas S. Ultrastructural analysis of the dehydrated tardigrade Hypsibius exemplaris unveils an anhydrobiotic-specific architecture. Sci Rep. 2020;10(1). 10.1038/s41598-020-61165-1 PMC7062702 32152342

[44] Vitkova VP, Alexander G (2013). Lipid bilayers and membranes: material properties. Advances in planar lipid bilayers and liposomes.

[45] Lewis RNAH, McElhaney RN (2013). Membrane lipid phase transitions and phase organization studied by Fourier transform infrared spectroscopy. Biochim Biophys Acta Biomembr.

[46] Disalvo EA, Bouchet AM, Frias MA (2013). Connected and isolated CH2 populations in acyl chains and its relation to pockets of confined water in lipid membranes as observed by FTIR spectrometry. Biochim Biophys Acta Biomembr.

[47] Rosa AS, Cejas JP, Disalvo EA, Frías MA (2019). Correlation between the hydration of acyl chains and phosphate groups in lipid bilayers: effect of phase state, head group, chain length, double bonds and carbonyl groups. Biochim Biophys Acta Biomembr.

[48] Roy A, Dutta R, Kundu N, Banik D, Sarkar N (2016). A comparative study of the influence of sugars sucrose, Trehalose, and maltose on the hydration and diffusion of DMPC lipid bilayer at complete hydration: investigation of structural and spectroscopic aspect of lipid–sugar interaction. Langmuir.

[49] Cacela C, Hincha DK (2006). Low amounts of sucrose are sufficient to depress the phase transition temperature of dry Phosphatidylcholine, but not for Lyoprotection of liposomes. Biophys J.

[50] Kent B, Hauß T, Demé B, Cristiglio V, Darwish T, Hunt T, Bryant G, Garvey CJ (2015). Direct comparison of disaccharide interaction with lipid membranes at reduced hydrations. Langmuir.

[51] Popova AV, Hundertmark M, Seckler R, Hincha DK (2011). Structural transitions in the intrinsically disordered plant dehydration stress protein LEA7 upon drying are modulated by the presence of membranes. Biochim Biophys Acta Biomembr.

[52] Navarro-Retamal C, Bremer A, Ingólfsson HI, Alzate-Morales J, Caballero J, Thalhammer A, González W, Hincha DK (2018). Folding and lipid composition determine membrane interaction of the disordered protein COR15A. Biophys J.

[53] Moore DS, Hansen R, Hand SC (2016). Liposomes with diverse compositions are protected during desiccation by LEA proteins from Artemia franciscana and trehalose. Biochim Biophys Acta Biomembr.

[54] Tolleter D, Hincha DK, Macherel D (2010). A mitochondrial late embryogenesis abundant protein stabilizes model membranes in the dry state. Biochim Biophys Acta Biomembr.

[55] Uversky VN (2013). Unusual biophysics of intrinsically disordered proteins. Biochim Biophys Acta, Proteins Proteomics.

[56] Theillet F-X, Binolfi A, Frembgen-Kesner T, Hingorani K, Sarkar M, Kyne C, Li C, Crowley PB, Gierasch L, Pielak GJ (2014). Physicochemical properties of cells and their effects on intrinsically disordered proteins (IDPs). Chem Rev.

[57] Riback JA, Bowman MA, Zmyslowski A, Knoverek CR, Jumper J, Kaye EB, Freed KF, Clark PL, Sosnick TR (2018). Response to comment on “innovative scattering analysis shows that hydrophobic disordered proteins are expanded in water”. Science.

[58] Salvi N, Abyzov A, Blackledge M (2019). Solvent-dependent segmental dynamics in intrinsically disordered proteins. Sci Adv.

[59] Furuki T, Shimizu T, Chakrabortee S, Yamakawa K, Hatanaka R, Takahashi T, Kikawada T, Okuda T, Mihara H, Tunnacliffe A (2012). Effects of Group 3 LEA protein model peptides on desiccation-induced protein aggregation. Biochim Biophys Acta, Proteins Proteomics.

[60] Furuki T, Takahashi Y, Hatanaka R, Kikawada T, Furuta T, Sakurai M (2020). Group 3 LEA protein model peptides suppress heat-induced lysozyme aggregation. Elucidation of the underlying mechanism using coarse-grained molecular simulations. J Phys Chem B.

[61] Furuki T, Sakurai M (2014). Group 3 LEA protein model peptides protect liposomes during desiccation. Biochim Biophys Acta Biomembr.

[62] Shimizu T, Kanamori Y, Furuki T, Kikawada T, Okuda T, Takahashi T, Mihara H, Sakurai M (2010). Desiccation-induced structuralization and glass formation of group 3 late embryogenesis abundant protein model peptides. Biochemistry.

[63] Wang G, Bonkovsky HL, De Lemos A, Burczynski FJ (2015). Recent insights into the biological functions of liver fatty acid binding protein 1. J Lipid Res.

[64] Amiri M, Yousefnia S, Seyed Forootan F, Peymani M, Ghaedi K, Nasr Esfahani MH (2018). Diverse roles of fatty acid binding proteins (FABPs) in development and pathogenesis of cancers. Gene.

[65] Hotamisligil GS, Bernlohr DA (2015). Metabolic functions of FABPs—mechanisms and therapeutic implications. Nat Rev Endocrinol.

[66] Nishimoto T, Takahashi Y, Miyama S, Furuta T, Sakurai M (2019). Replica exchange molecular dynamics simulation study on the mechanism of desiccation-induced structuralization of an intrinsically disordered peptide as a model of LEA proteins. Biophys Physicobiol.

[67] Bruni F, Leopold AC (1991). Glass transitions in soybean seed : relevance to anhydrous biology. Plant Physiol.

[68] Crowe JH, Carpenter JF, Crowe LM (1998). The role of vitrification in Anhydrobiosis. Annu Rev Physiol.

[69] Hengherr S, Worland MR, Reuner A, Brümmer F, Schill RO (2009). High-temperature tolerance in anhydrobiotic tardigrades is limited by glass transition. Physiol Biochem Zool.

[70] Wright JC (1989). Desiccation tolerance and water-retentive mechanisms in Tardigrades. J Exp Biol.

[71] Blasi P, D'Souza SS, Selmin F, Deluca PP (2005). Plasticizing effect of water on poly(lactide-co-glycolide). J Control Release.

[72] Farahnaky A, Badii F, Farhat IA, Mitchell JR, Hill SE (2005). Enthalpy relaxation of bovine serum albumin and implications for its storage in the glassy state. Biopolymers.

[73] Clegg J (1978). Hydration-dependent metabolic transitions and the state of cellular water in Artemia cysts.

[74] Clegg J. The physical properties and metabolic status of Artemia cysts at low water contents: the ‘water replacement hypothesis’. Membr Metab Dry Organ. 1986:169–87.

[75] Potts M (1994). Desiccation tolerance of prokaryotes. Microbiol Rev.

[76] Hill JJ, Shalaev EY, Zografi G (2014). The importance of individual protein molecule dynamics in developing and assessing solid state protein preparations. J Pharm Sci.

[77] Giuffrida S, Cordone L, Cottone G (2018). Bioprotection can be tuned with a proper protein/saccharide ratio: the case of solid amorphous matrices. J Phys Chem B.

[78] Crowe LM (2002). Lessons from nature: the role of sugars in anhydrobiosis. Comp Biochem Physiol A Mol Integr Physiol.

[79] Fedorov MV, Goodman JM, Nerukh D, Schumm S (2011). Self-assembly of trehalose molecules on a lysozyme surface: the broken glass hypothesis. Phys Chem Chem Phys.

[80] Cordone L, Cottone G, Cupane A, Emanuele A, Giuffrida S, Levantino M (2015). Proteins in saccharides matrices and the Trehalose peculiarity: biochemical and biophysical properties. Curr Org Chem.

[81] Bellavia G, Giuffrida S, Cottone G, Cupane A, Cordone L (2011). Protein thermal denaturation and matrix glass transition in different protein−Trehalose−water systems. J Phys Chem B.

[82] Lerbret A, Affouard F, Hédoux A, Krenzlin S, Siepmann J, Bellissent-Funel M-C, Descamps M (2012). How strongly does Trehalose interact with lysozyme in the solid state? Insights from molecular dynamics simulation and inelastic neutron scattering. J Phys Chem B.

[83] Lerbret A, Affouard F, Bordat P, Hédoux A, Guinet Y, Descamps M (2011). Slowing down of water dynamics in disaccharide aqueous solutions. J Non-Cryst Solids.

[84] Giuffrida S, Cottone G, Cordone L (2017). Water association band as a marker for hydrogen bond in trehalose amorphous matrices. Phys Chem Chem Phys.

[85] Das RK, Ruff KM, Pappu RV (2015). Relating sequence encoded information to form and function of intrinsically disordered proteins. Curr Opin Struct Biol.

[86] Das RK, Pappu RV (2013). Conformations of intrinsically disordered proteins are influenced by linear sequence distributions of oppositely charged residues. Proc Natl Acad Sci.

[87] Holehouse AS, Das RK, Ahad JN, Richardson MOG, Pappu RV (2017). CIDER: resources to analyze sequence-ensemble relationships of intrinsically disordered proteins. Biophys J.

[88] Janis B, Belott C, Menze MA (2018). Role of intrinsic disorder in animal desiccation tolerance. Proteomics.

[89] Kamilari M, Jørgensen A, Schiøtt M, Møbjerg N. Comparative transcriptomics suggest unique molecular adaptations within tardigrade lineages. BMC Genomics. 2019;20(1). 10.1186/s12864-019-5912-x PMC6652013 31340759

[90] Brown CJ, Johnson AK, Dunker AK, Daughdrill GW (2011). Evolution and disorder. Curr Opin Struct Biol.

[91] Huang H, Sarai A (2012). Analysis of the relationships between evolvability, thermodynamics, and the functions of intrinsically disordered proteins/regions. Comput Biol Chem.

